# Technologies and main functionalities of the telemonitoring application reCOVeryaID

**DOI:** 10.3389/fdata.2024.1360092

**Published:** 2024-07-22

**Authors:** Daniela D'Auria, Fabio Bettini, Selene Tognarelli, Diego Calvanese, Arianna Menciassi

**Affiliations:** ^1^Faculty of Engineering, Free University of Bozen-Bolzano, Bolzano, Italy; ^2^The BioRobotics Institute, Scuola Superiore Sant'Anna, Pisa, Italy; ^3^Department of Computing Science, Umeå University, Umeå, Sweden

**Keywords:** artificial intelligence, coronavirus, COVID-19, eHealth, long-term monitoring, rule-based system, telehealth, telemedicine

## Abstract

The COVID-19 pandemic has highlighted the need to take advantage of specific and effective patient telemonitoring platforms, with specific reference to the constant monitoring of vital parameters of patients most at risk. Among the various applications developed in Italy, certainly there is reCOVeryaID, a web application aimed at remotely monitoring patients potentially, currently or no longer infected with COVID-19. Therefore, in this paper we present a system model, consisting of a multi-platform intelligent telemonitoring application, that enables remote monitoring and provision of integrated home care to both patients symptomatic, asymptomatic and pre-symptomatic with severe acute respiratory infectious disease or syndrome caused by viruses belonging to the Coronavirus family, as well as simply to people with respiratory problems and/or related diseases (chronic obstructive pulmonary disease or asthma). In fact, in this paper we focus on exposing the technologies and various functionalities offered by the system, which constitute the practical implementation of the theoretical framework described in detail in another paper. Specifically, the reCOVeryaID telemonitoring application is a stand-alone, knowledge base-supported application that can promptly react and inform physicians if dangerous trends in a patient's short- and long-term vital signs are detected, thus enabling them to be monitored continuously, both in the hospital and at home. The paper also reports an evaluation of user satisfaction, carried out by actual patients and medical doctors.

## 1 Introduction

SARS-CoV-2, a member of the coronavirus family, is the virus that causes COVID-19, an *infectious respiratory disease*. Following a flu-like early phase, a very severe respiratory condition linked to the development of bilateral interstitial pneumonia may emerge later in the disease. Breathlessness, dyspnea, difficulty breathing, and elevated heart rate are some of its symptoms. In actuality, until the need for hospitalization becomes urgent, COVID-19 pneumonia causes a drop in blood oxygen saturation without the patient's awareness. Because of this, it is essential to keep an eye on people who are being monitored for COVID-19 infection at home to make sure their saturation level does not drop below the set threshold especially if they have not had any prior respiratory illnesses. Medical personnel can determine whether to hospitalize a patient who is asymptomatic, symptomatic, or pre-symptomatic and receiving home isolation by trending the saturation data. This allows for an *early hospitalization* before the patient's clinical picture deteriorates. Thus, the creation of an intelligent telemonitoring system that makes use of conventional diagnostic tools like an oximeter and thermometer will enable to satisfy the following constraints: *(i)* those who are not in immediate danger should be left at home, saving hospital beds; *(ii)* watch those who could experience a respiratory crisis; *(iii)* keep an eye on the portion of the population (such as those under precautionary quarantine) that has not been tested for COVID-19 but may be asymptomatic or pre-symptomatic; *(iv)* assess critical factors to prevent a respiratory crisis in individuals with mild COVID-19; *(v)* enable general practitioners to monitor the patient round-the-clock, reducing the possibility of infections from prolonged and/or direct contact.

In order to achieve these goals, we designed and developed *reCOVeryaID* (D'Auria et al., 2023b), an intelligent telemonitoring application for symptomatic, asymptomatic, and pre-symptomatic coronavirus patients; in this paper, starting from the theoretical model shown in D'Auria et al. (2023b), we focus on highlighting its technologies and main functionalities, pointing out that a wide application of this framework in Italian hospitals could be really essential for both the health care system and the population. Specifically, *reCOVeryaID* is a web application aimed at remotely monitoring patients potentially, currently or no longer infected with COVID-19; it consists of a prototype implementation allowing patients and medical doctors to effectively interact with each other, to cope with the issues listed above. The necessary equipment consists of a saturimeter for measuring oxygen saturation (SpO_2_) and heart rate, a thermometer for measuring body temperature the *reCOVeryaID* web-app, the patient's smartphone (or laptop), and the doctor's smartphone (or laptop). The carried out measurements are the SpO_2_ value, the body temperature value, and the heart rate value. Eventually, the framework returns both short-term and long-term alerts, which need to be further investigated by medical doctors. In this way, patients, especially the frail ones, can be constantly monitored by the app, which provides prompt alerts to the physician if abnormal trends in vital parameters are identified in both the short and long term.

The remainder of the paper is organized as follows. In the next section we show the related work in the eHealth context, with particular focus on monitoring vital signs. Then, Section 3 illustrates in detail the used technologies, both from the client side and from the server side, and the overall application behavior. Additionally, Section 4 shows the framework functionalities, and Section 5 exposes the experimental evaluation, which concerns user satisfaction. Eventually, Section 6 concludes the paper and outlines future work.

## 2 Related work

Based on the current pandemic scenario and events that have transpired since the epidemic's onset, it is evident that telemedicine has not really taken off in Italy or the rest of the world. Aside from the clinical benefit, it might have saved a significant amount of money for a number of health systems, and it might have been very helpful in making primary care physicians' monitoring of COVID-19 patients and not just patients more dependable and manageable right from the start of the pandemic (Charles, 2000). On the other hand, telemedicine has tremendous potential, as this is highlighted by the following papers that precede or coincide with the first part of the pandemic (Ali and Khoja, 2020; Chauhan et al., 2020; Hollander and Carr, 2020; Monaghesh and Hajizadeh, 2020). This is demonstrated by the fragmented trials that are currently taking place in various parts of the world, as well as by the rise of startups, tools, and practical technologies for remote monitoring; additionally, health insurance companies are showing interest in this, as they are now including teleconsultations and far more advanced solutions in their fee-for-service packages; several national health systems could gain from wearable technology to virtual triage, from remote examinations (at least those that are practical) to vital sign monitoring.

The various Rheumatology Societies have also recently raised the alarm, or rather pushed for the use of telemedicine systems, citing the need for constant monitoring and prompt diagnosis and treatment for patients with lupus, rheumatoid arthritis, vasculitis, and other similar chronic and frequently autoimmune diseases; consequently, the papers (Danhieux et al., 2020; Dimitroulas and Bertsias., 2020; Mason et al., 2020; Wright et al., 2020; Coupet et al., 2021; Hacker et al., 2021) point out that the use of telemedicine is extremely useful not only to monitor the COVID-19 itself, but also to control the evolution of other types of pathologies, such as chronic diseases. They businesses actually plan to make significant investments in telemedicine, and several of them have already created online resources specifically for patients with rheumatology, but they are limited in scope. All diseases should be treated using a comparable model that is structured and applied globally.

We realize that the road to digitizing public health is still long, given that, for example, in Italy, where we had one of the largest pandemic spikes in the world at the beginning of the first pandemic wave, even electronic medical records, a relatively simple tool for coordinating all the services provided to citizens, never took off. Even with the mandated acceleration of the pandemic, only dematerialized prescriptions have become widely used thus far, limiting telemedicine mostly to general practitioners' (often busy) phones.

Various attempts have been made to mitigate the deficiency of sufficient telemedicine networks by employing artificial intelligence (AI) to forecast illness or diagnose patients (Li et al., 2020). Specifically, the goal of using AI combined with telemedicine is to provide the added value regarding future prediction in the context of numerous diseases. In fact, it has been suggested that artificial intelligence be used to evaluate chest CT scans (Han et al., 2020) and diagnose COVID-19 early. It has also been suggested to identify predictive markers for cytokine storm (Caricchio et al., 2021) or evaluate particular laboratory parameters (like lymphocyte count or hsCRP) to forecast the patient's worsening clinical condition. The primary issue with this study, aside from the challenge of validating new criteria, is that patients must undergo intrusive “screening” procedures, which include blood samples and CT scans, requiring hospitals and laboratories, and therefore increasing the expense of the diagnosis process. In order to avoid intervening until it is too late and the patient has already required hospitalization, other authors have proposed algorithms to predict the risk of death in hospitalized patients (Gao et al., 2020) and the necessity for invasive ventilation (Burdick et al., 2020).

In addition to the aforementioned information and in line with earlier research from multiple studies, it is evident that using an oximeter to monitor saturation level to follow COVID-19 patients is essential, as highlighted by the following papers (Taguchi et al., 1994; Solé et al., 2009; Scott and McDougall, 2017; Elliott and Baird, 2019; Takei et al., 2020); specifically, they need to be closely watched to ensure that their saturation level does not drop below a certain point, particularly if they have not previously experienced a respiratory illness. Medical personnel can determine whether to admit a patient who is asymptomatic, symptomatic, or pre-symptomatic and is receiving home isolation by analyzing trending saturation data. This allows them to make an early admission before the patient's clinical condition deteriorates.

As a result of the discussion conducted so far, we point out that all of the listed issues can be resolved by an integrated home care/telemedicine system exploiting many devices, including a saturation device and a thermometer; specifically, an algorithm examines and filters the data, presenting any alarms to the referring medical doctor in real time. Alternatively, the patient could supply the data required for analysis on their own. The benefits will include avoiding invasive testing like CT scans and blood draws, as well as a decrease in hospital stays for less severe symptoms that do not need for extensive care. The following is a list of other relevant and recent telemonitoring systems that use vital parameter monitoring: (Wiffen et al., 2023) facilitates the improvement of data collection and processing in order to create a reliable app that can be used on a daily basis in clinical settings, while (Ko et al., 2023) performed in-person home visits once a day, with nursing visits up to three times a day for intravenous therapy; patients were also released from the program when they satisfied standard inpatient discharge criteria. Murali (2023) provide a more efficient method of monitoring the patient's healthcare system. Their primary areas of interest are cloud services, IoT, and machine learning (ML) in the context of patient monitoring. Furthermore, their technology may be utilized as an advanced Internet of Things-enabled real-time patient monitoring system to monitor the patient's vital indicators, including blood pressure, heart rate, oxygen saturation, and body temperature. Subsequently, Totuk et al. (2023) examined Value Stream Mapping (VSM) measures using smartphones, pulse oximetry probes, and blood group antigens (BGA). The VSM, smartphone, and BGA all shown very good agreement in the Integrated Comprehensive Care (ICC) of the oxygen saturation of arterial blood (SaO_2_) readings. Similar to this, there was excellent agreement between the VSM and smartphone in terms of the ICC value of the Heart Rate (HR) values. Moreover, according to Smith et al. (2021), the success of wearable technologies in the future depends on developing clinical confidence in the accuracy of the data measured and the appropriate interpretation of that data in relation to the person, the environment, and the activity carried out. In fact, wearable physiological monitoring may soon enhance point-of-care diagnostic accuracy and provide crucial information for medical decisions.

In this context, the proposed framework *reCOVeryaID* leverages an innovative combination of (wearable) devices to generate short-term and long-term alerts related to COVID-19; additionally, the *reCOVeryaID* prototype's flexibility and generality allow it to be combined with other systems that address various diseases, which is another strength of the system; indeed, it might be combined with complex event processing and intelligent agent-based telemonitoring systems (Persia et al., 2021; De Lauretis et al., 2023), Italian clinical notes (D'Auria et al., 2023a), and a multi-agent system for epilepsy prediction and detection in neuropediatrics (Bertoncelli et al., 2023).

In order to improve browsing of related works, Table 1 summarizes them, listing first authors, year, and main topic.

**Table 1:** Summary of related work.

**First author**	**Year**	**Topic**
Ali	2020	The importance of telemedicine in addressing COVID-19
Bertoncelli	2023	A multi-agent based system for epilepsy detection and prediction in neuropediatrics
Burdick	2020	Prediction of respiratory decompensation in COVID-19 patients using machine learning
Caricchio	2021	Developing predictive criteria for COVID-19-associated cytokine storm
Charles BL	2020	The importance of telemedicine
Chauhan	2020	The importance of telemedicine in addressing COVID-19
Coupet	2021	Managing chronic disease during COVID-19
Danhieux	2020	The impact of COVID-19 on chronic care - case study in Belgium
D'Auria	2023b	Telemonitoring application for COVID-19 patients
D'Auria	2023a	Improving graph embeddings via entity linking: a case study on Italian clinical notes
De Lauretis	2023	Intelligent agents and complex event processing to improve patient monitoring
Dimitroulas	2020	Managing systemic inflammatory disorders during the COVID-19 pandemic
Elliott	2019	Pulse oximetry and the enduring neglect of respiratory rate assessment
Gao	2020	Machine learning based early warning system
Hacker	2021	COVID-19 and chronic disease
Han	2023	Accurate screening of COVID-19 using attention-based deep learning
Hollander	2020	The importance of telemedicine in addressing COVID-19
Ko	2023	Continuous vital signs monitoring in patients hospitalized at home
Li	2020	Using machine learning of clinical data to diagnose COVID-19
Mason	2020	Clinical management of Lupus patients during the COVID-19 pandemic
Monaghesh	2020	The role of telehealth during COVID-19 outbreak
Murali	2023	Machine learning and internet of things to improve patient health monitoring systems
Persia	2021	A smart framework for automatically analyzing electrocardiograms
Scott	2017	The effective introduction of Lifebox pulse oximetry to Malawi
Smith	2020	Multimodal biosensor for remote physiological monitoring
Solé	2009	Pulse oximetry in the evaluation of the severity of acute asthma in children
Taguchi	1994	How to assess bronchial asthma and desaturation with pulse oximetry
Takei	2019	Prediction of prognosis of idiopathic pulmonary fibrosis by pulse oximetry saturation
Totuk	2023	Reliability of smartphone measurements of peripheral oxygen saturation and heart rate
Wiffen	2023	Measurement of vital signs by Lifelight software
Wright	2020	Neglected chronic disease management during COVID-19

## 3 The used technologies

**Figure 1:**
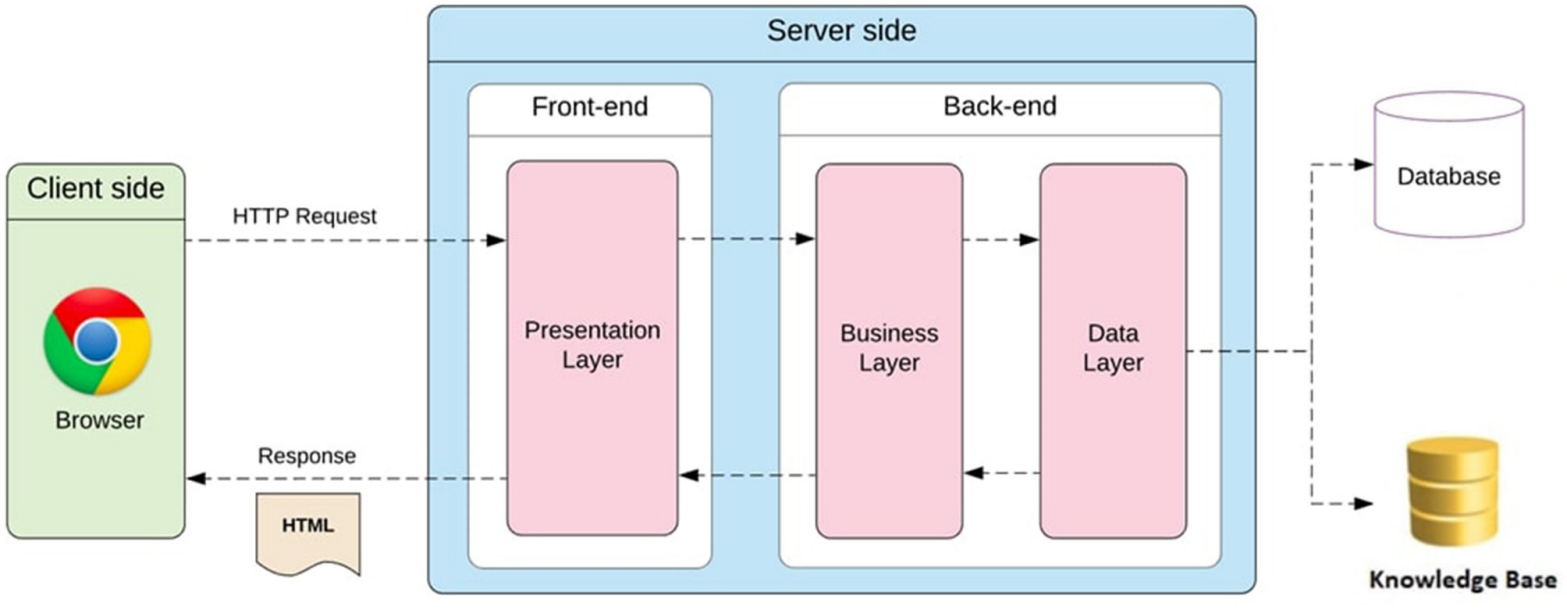
The framework architecture.

Figure 1 shows the architecture of the proposed framework. Specifically, the technologies used to implement the reCOVeryaID cross-platform application can be classified into *client-side* technologies and *server-side* technologies.

### 3.1 Client-side technologies

The client-side technologies employed in the realization of the reCOVeryaID cross-platform application were basically two:

- *Flutter*^1^: Google's open source framework for creating cross-platform (Android, iOS, Web, Linux, Windows, MacOS, Embedded) applications compiled natively from a single code base;
- *Dart*^2^: open source language supported by Google and optimized to have fast applications on any kind of platform (Android, iOS, Web, Linux, Windows, MacOS, Embedded).

Additionally, the following tools are needed:

- An oximeter that the patient can use at home to test oxygen saturation (SpO_2_);
- A body temperature thermometer that the patient can use at their own house;
- The patient's computer or smartphone;
- The physician's computer or smartphone;
- The free reCOVeryaID Web-App.

### 3.2 Server-side technologies

The server-side technologies employed in the realization of the reCOVeryaID cross-platform application were basically three:

- The database;
- The web server;
- A web folder.

The Database is a MySQL 8.0.27-0ubuntu0.20.04.1 with phpMyAdmin 4.9.5deb2 via UNIX socket. The Web Server is an Apache/2.4.41-Ubuntu with PHP 7.4.3. Additionally, the Web Folder uses the WebDAV protocol.

All the knowledge-base rules have been implemented as triggers or stored procedures within the MySQL database management system.

### 3.3 The application behavior

In particular, the app allows general practitioners or other medical specialists to keep an eye on patients based on measures that the patient submits via the app. *Heart rate (HR), blood pressure (*SpO_2_*), body temperature (BT)*, and *the associated timestamp* must all be included in each measurement. The system will designate an alert level (*red, yellow*, or *green*) for each measurement based on rules kept in a particular knowledge base. It was decided to employ *threshold values* of temperature, SpO_2_, and HR that have been validated by medical professionals in the field while designing the rules to be adopted to generate warnings based on the data taken by the patient. At this point, the physician can simply respond to the patient with the proper feedback message after receiving the measurement and its alert level and viewing (with ease via the Web-App) the historical trend of the various measurements. This message will be:

- In the event of a green alert, an OK;
- Particular instructions to the patient in the event of a yellow alert;
- A warning that an ambulance will be responding in the event of a red alert.

In the final scenario, the application will, pending physician confirmation, dial an ambulance. Furthermore, the system will run more thorough statistical evaluations of each patient's most recent *N* measurements on a regular basis. These checks will be kept in a database that will track *patient ID, measurement interval*, and *outcome*. It may also produce additional alerts that are linked to a longer time interval rather than the most recent timestamp. The system will indicate a *long-term anomaly* of the concerned parameter, for instance, if *M* measurements out of the last *N* (with *M* ≤ *N*) are too near to the red threshold of one of the three critical parameters. As of right now, *reCOVeryaID* is a prototype. The system is novel because of its rapid and simple protocol for patient-physician contact and its unique set of criteria involving important parameters that are kept in the knowledge base and are intended to trigger alarms. Considering these unique characteristics, *reCOVeryaID* can be seamlessly applied to other domains not exclusively associated with COVID-19 emergencies, like tracking patients with conditions like diabetes or high blood pressure, thereby expanding its capabilities and fortifying physicians' acceptance of it.

**Figure 2:**
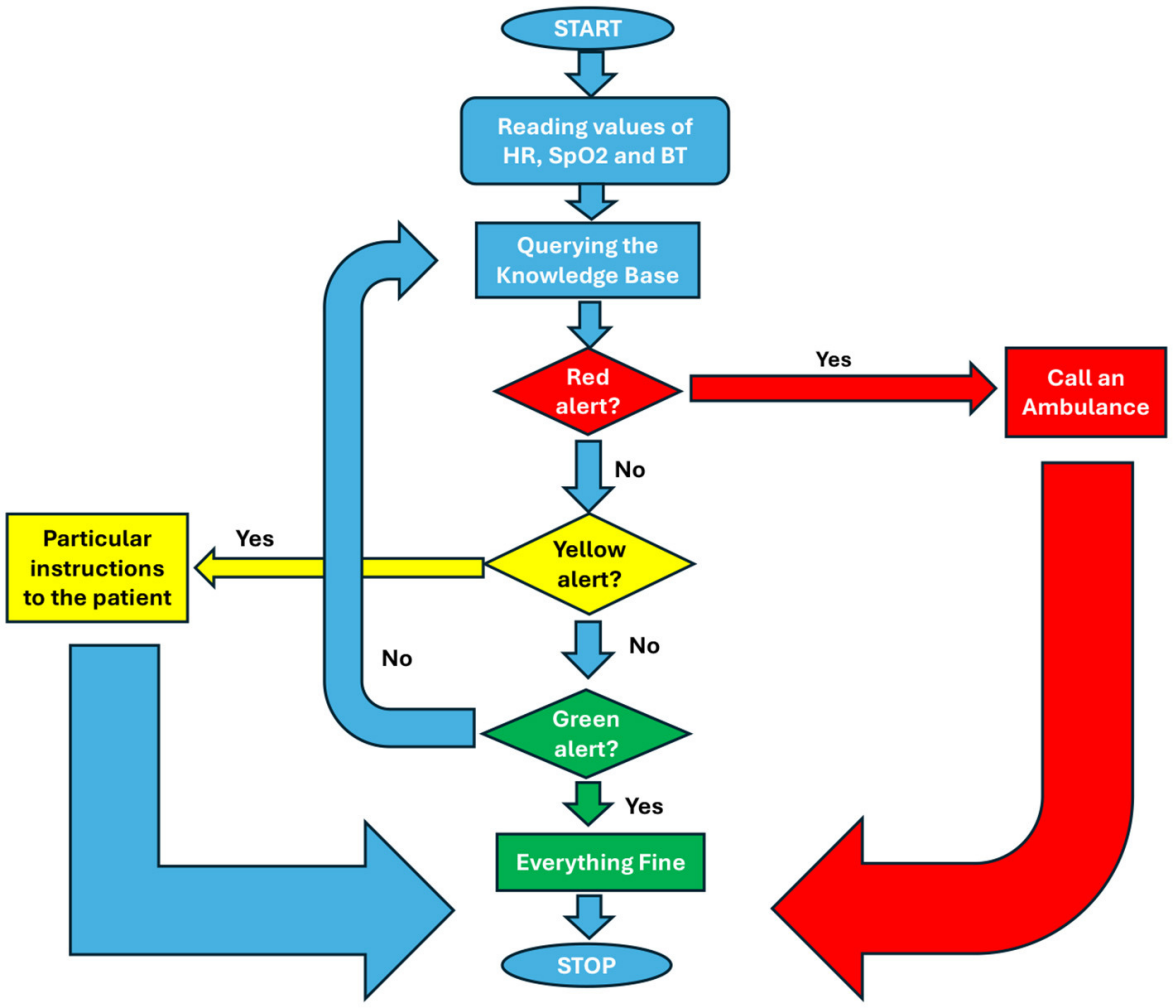
Flow chart diagram showing the short-term alert generation process.

In summary, Figure 2 highlights the system behavior when generating short-term alerts.

**Figure 3:**
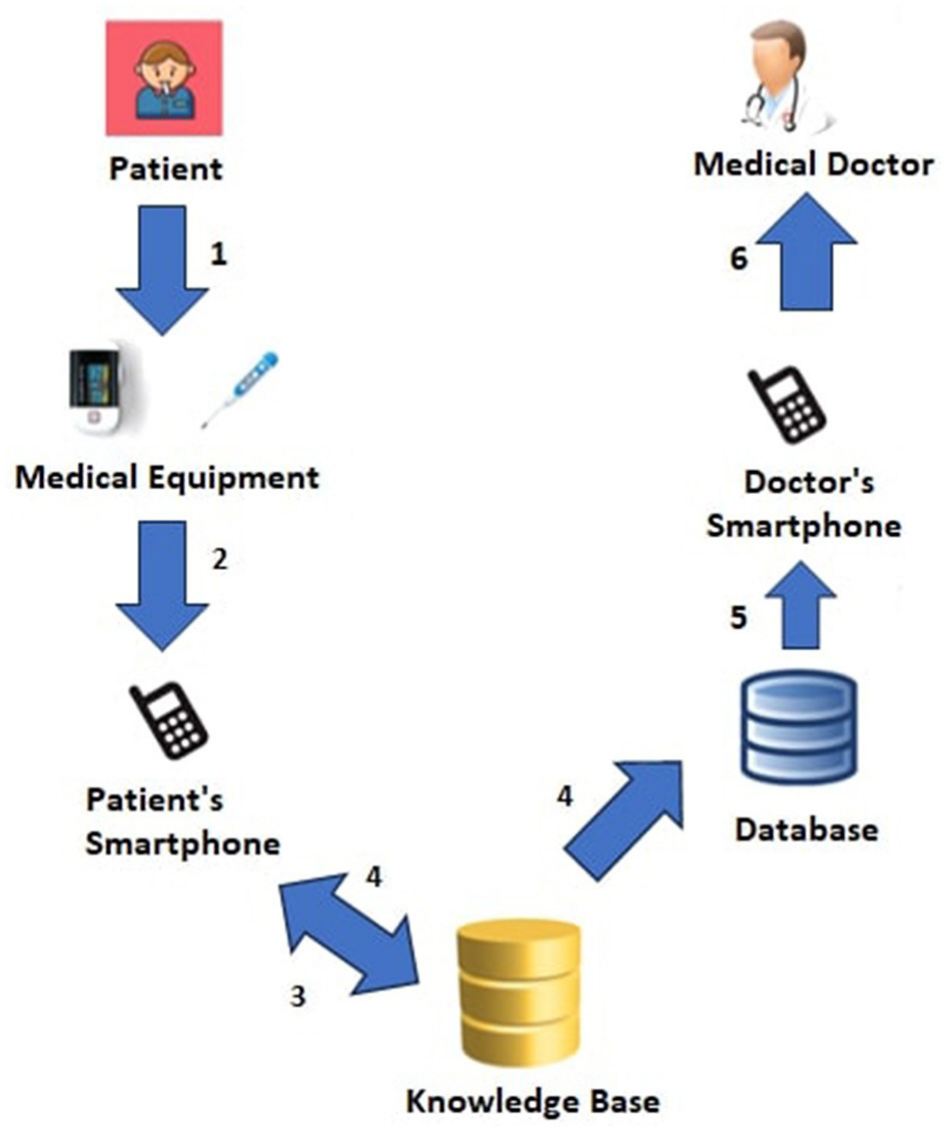
*Patient*→*Medical Doctor* interaction protocol.

Additionally, Figure 3 shows more details about the *Patient*→*Medical Doctor Interaction Protocol*; specifically, it consists of the following steps:

1. Using the *oximeter* and *thermometer*, two pieces of medical equipment, the patient (regularly) monitors his or her vital parameters.
2. The patient's smartphone receives the current measurement (heart rate, oxygen saturation, and temperature) either automatically using Bluetooth or manually.
3. The knowledge base computes the associated the *short-term* alert by analyzing the current measurement, and *long-term* alerts by analyzing the current measurement and the previous *N* ones.
4. The patient is shown the measurement and the associated short- and long-term alerts right away; additionally, and short- and long-term alerts are saved in the database.
5. The doctor's smartphone receives the calculated alerts.
6. All notifications, ranked from *red* to *green* based on urgency, are accessible to the medical professional.

**Figure 4:**
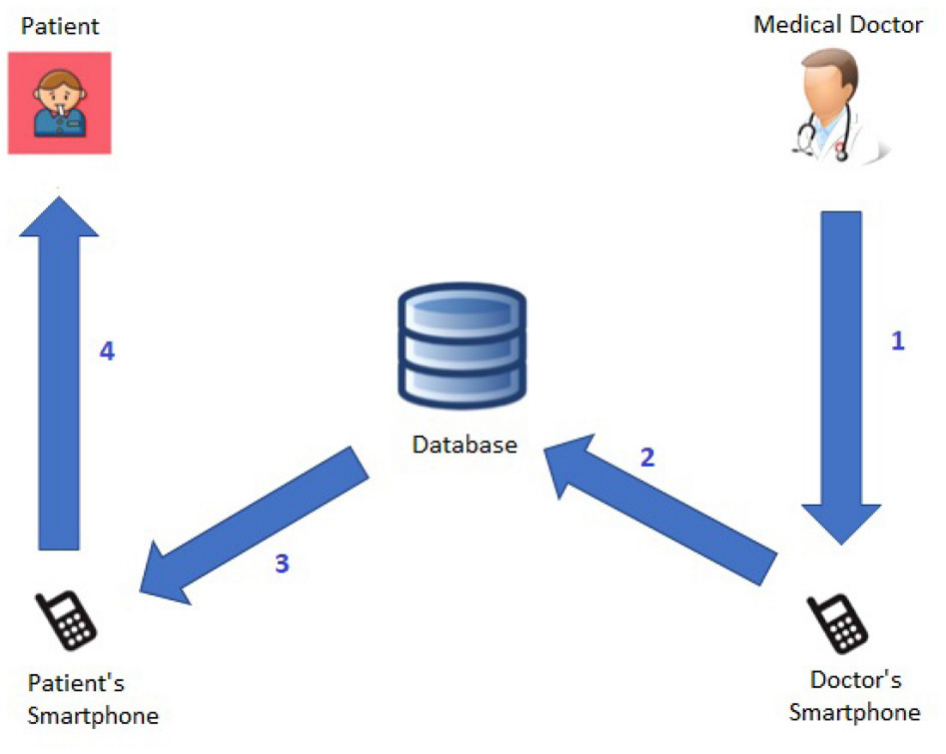
*Medical Doctor*→*Patient* Interaction protocol.

Moreover, Figure 4 shows more details about the *Medical Doctor*→*Patient Interaction Protocol*, which consists of the following steps:

1. The patient receives a feedback message from the medical professional after examining the patient's short- and long-term alerts via the smartphone.
2. The feedback message is kept in the database by the system.
3. The patient's smartphone receives the feedback message from the system.
4. The patient can view the feedback message from the medical doctor.

## 4 The framework functionalities

The functionalities provided by the multi-platform *reCOVeryaID* application are: *system registration and user account creation* (Section 4.1), *user account activation* (Section 4.2), *password retrieval* (Section 4.3), *login* (Section 4.4), *log off* (Section 4.5), *homepage* (Section 4.6), *user profile* (Section 4.7), *new measurement* (Section 4.8), *measurement history* (Section 4.9), *long-term alert history* (Section 4.10), *settings* (Section 4.11), and *information* (Section 4.12).

### 4.1 System registration and user account creation

The “system registration and user account creation” feature (Supplementary Figure 1) allows the user to register to the system and create his or her own account by entering the e-mail, password, and password confirmation, but only after agreeing to the “terms and conditions” of using the application.

### 4.2 User account activation

The “user account activation” feature (Supplementary Figure 2) allows the user to activate the account, created during registration, by entering into the application the activation code that the system sends to the e-mail address provided during registration.

### 4.3 Password retrieval

The “password recovery” feature (Supplementary Figure 3A) allows users to recover access to their account by entering the e-mail provided during registration, the new password, and the new password confirmation.

### 4.4 Login

The “login” feature (Supplementary Figure 3B) allows the user both to access the application (i.e., the features it provides), by entering the e-mail and password provided during registration, and to select the language of the application (Supplementary Figure 4A—currently English, Italian, and German are supported).

### 4.5 Log off

The “log off” feature (Supplementary Figure 4B) allows the user to log out of the application downstream, however, with a login and session still open in the system.

### 4.6 Homepage

The “homepage” feature (Supplementary Figure 5A) allows the user to view information from the last measurement (Supplementary Figure 5B)—temperature, heart rate, oxygen saturation, date and time taken and sent to the system and any long-term alerts (Supplementary Figures 6A, B, 7A, B).

It also allows both access to additional application screens (user profile, new measurement, measurement history, long-term alert history, settings, information) and logging out of the application (Supplementary Figure 4B).

### 4.7 User profile

The “user profile” feature (Supplementary Figure 8A) allows the user to view his or her personal profile, which currently consists only of the e-mail and password provided during registration.

### 4.8 New measurement

The “new measurement” feature (Supplementary Figure 8B) allows the user to provide a new measurement to be sent to the system. This measurement consists of both parameters (temperature, heart rate, oxygen saturation) and time information (date, time). The report of the new measurement, on the other hand, provides not only information about the new measurement just sent to the system, but also an indication of whether there are (Supplementary Figure 9A) or not (Supplementary Figure 9B) long-term alerts.

The operation can be done in two different ways:

- Manual.
- Automatic.

In the *manual mode*, the new measurement is provided by entering both parameter values and time information values; whereas, in the *automatic mode* the new measurement is provided by capturing both parameter values and time information values.

Specifically, the acquisition of the new measurement in the automatic mode is done using an external device with Bluetooth technology so as to facilitate data collection and at the same time have greater security and integrity of the measurements.

### 4.9 Measurement history

The “measurement history” feature (Supplementary Figure 10A) allows the user to view the complete list of measurements (Supplementary Figures 11, 12A) entered and sent to the system (or a subset if a time interval is specified) focused on all parameters (temperature, heart rate oxygen saturation) and with related graphical preview (Supplementary Figures 10B, 12, 13A for temperature—Supplementary Figures 13B, 14 for heart rate—Supplementary Figures 15, 16A for oxygen saturation) and/or related detailed graph (Supplementary Figures 16B, 17) if focused on a single parameter.

Specifically, for each measurement it is possible both to view its details (Supplementary Figure 29—temperature, heart rate, oxygen saturation, date, time -, Supplementary Figure 18A) and to make a final deletion (Supplementary Figure 18B) from the measurement history.

In addition, it can be exported via e-mail (Supplementary Figures 19, 20A) in order to continue with a medical consultation.

### 4.10 Long-term alert history

The “long-term alert history” feature (Supplementary Figure 20B) allows the user to view the complete list of long-term alerts (Supplementary Figure 21) that have been triggered in the system downstream of sending measurements (or a subset if a time interval is specified) focused separately on each parameter (temperature, heart rate, oxygen saturation).

Specifically, for each long-term alert, it is possible to view its details (Supplementary Figure 22 for temperature, Supplementary Figure 23 for heart rate, Supplementary Figure 24 for oxygen saturation)—duration interval of the alert, set of measurements that triggered it in turn detailed with the date, time and measurement.

### 4.11 Settings

The “settings” feature (Supplementary Figure 25A) allows the user to select the language of the application (Supplementary Figure 25B—currently English, Italian and German are supported) and the units of measurement for the parameters (Supplementary Figure 26A) that characterize the measurements:

- Temperature;
- Heart rate;
- Oxygen saturation.

Temperature (Supplementary Figure 26B) can be set in either degrees Celsius or degrees Fahrenheit, while heart rate (Supplementary Figure 27A) and oxygen saturation (Supplementary Figure 27B) are set by default in beats per minute (bpm) and percent (%), respectively.

In addition, you can both reset and/or change the password for accessing your account (Supplementary Figure 28A—functionality) and view information regarding the account deletion procedure.

### 4.12 Information

The “information” feature (Supplementary Figure 28B) allows the user to view “terms and conditions,” “privacy policy,” and the application web page.

In addition, you can contact the support team (Supplementary Figure 29) by e-mail.

## 5 Experimental evaluation

In order to validate the developed framework, we evaluated the impact of our system on different categories of users (i.e., *patients* and *medical doctors*) engaged in performing the tasks mentioned in Section 4. To evaluate the results, we used the TLX (NASA Task Load Index factor)^3^; more specifically, we asked users to express their opinions about the capability of our system to provide an effective user experience in completing all the tasks described in Section 4, based on the TLX evaluation protocol. In fact, TLX is a multidimensional rating procedure that provides a score from 1 to 5 on six subscales: *mental demand, physical demand, temporal demand, low performance, effort*, and *frustration*. Lower TLX scores are better.

**Figure 5:**
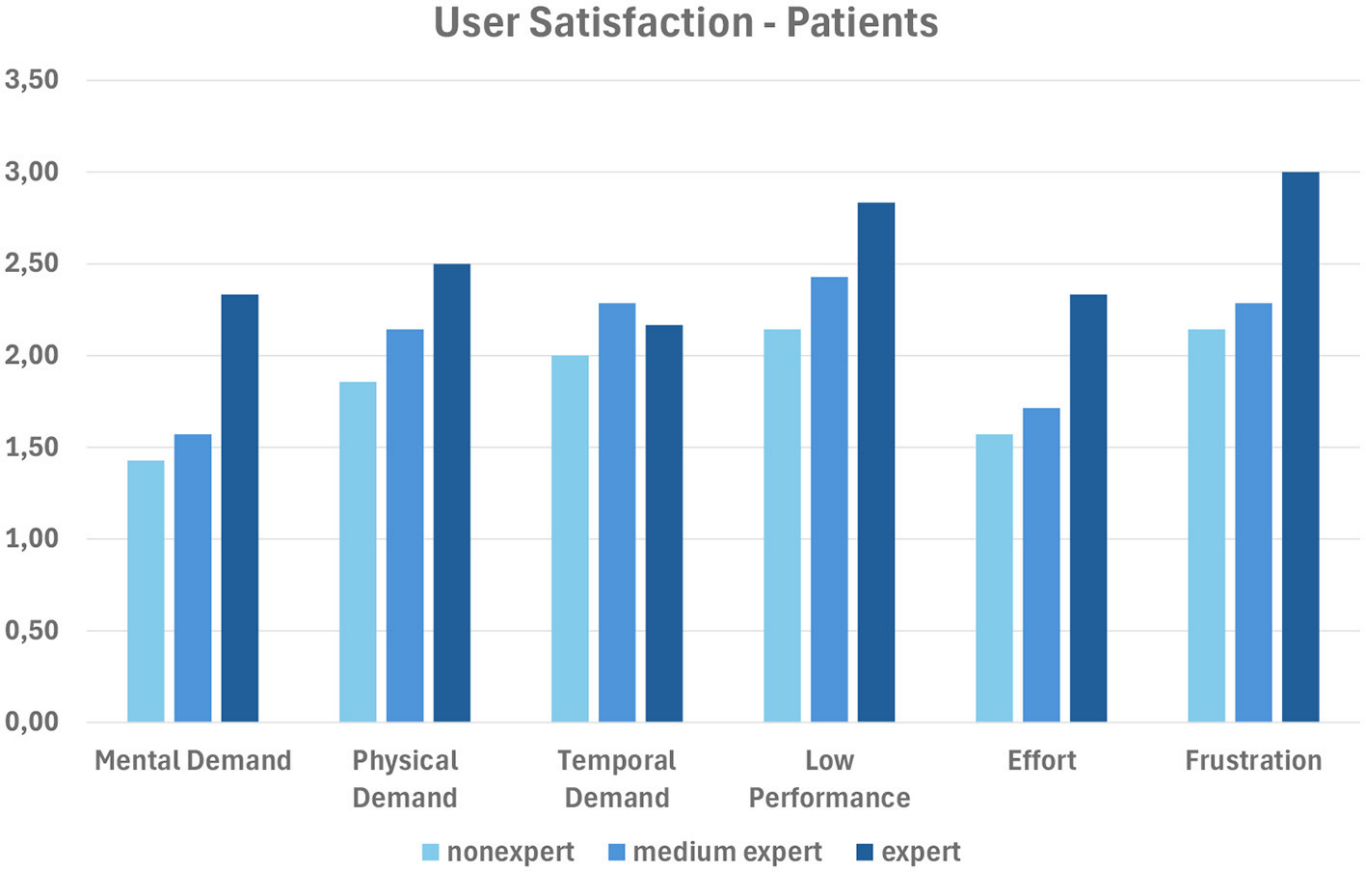
Average user satisfaction of patients.

**Figure 6:**
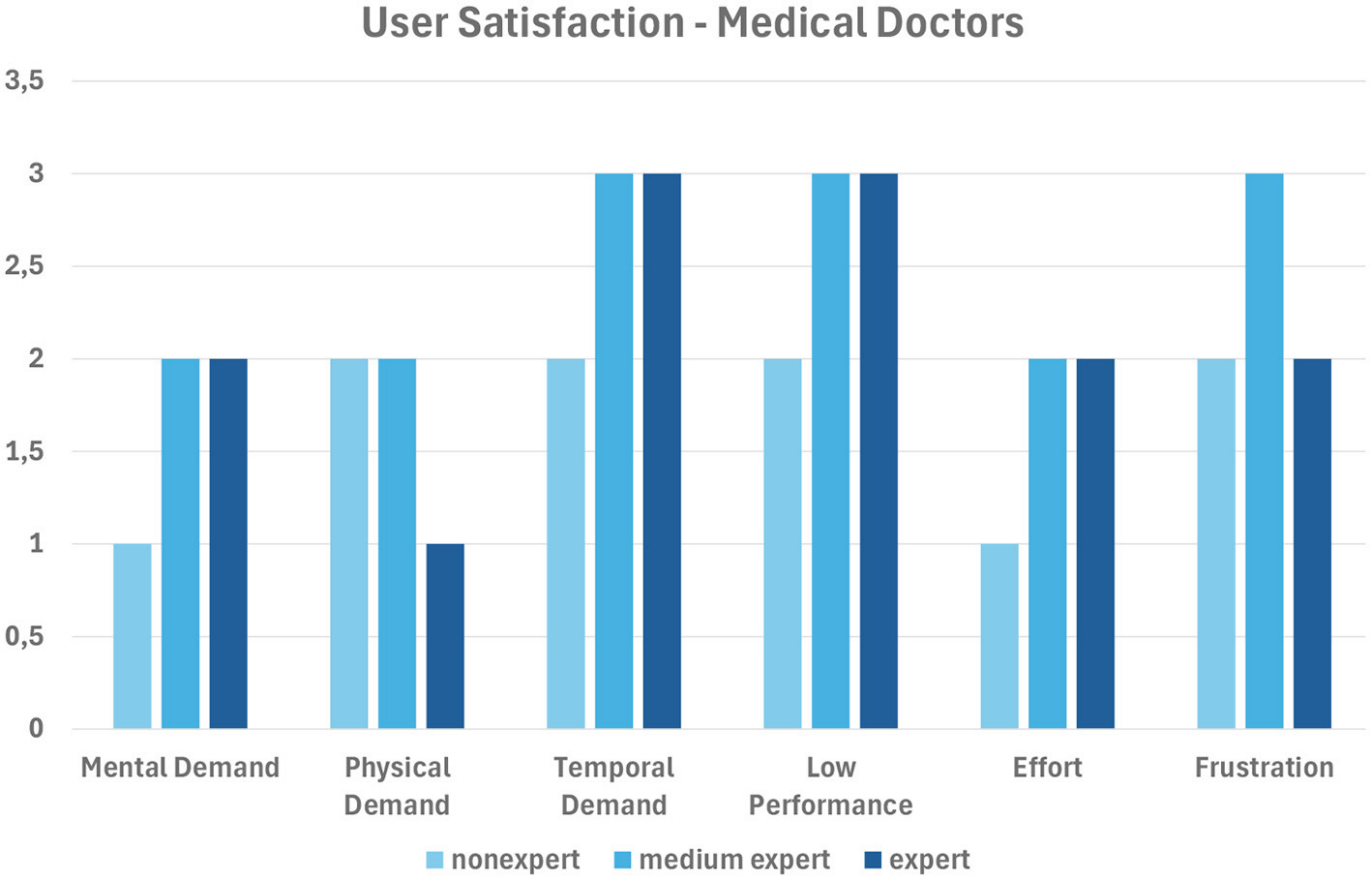
Average user satisfaction of medical doctors.

Figure 5 shows the scores achieved by each of the three categories of the 20 patient involved (*expert, medium expert*, and *nonexpert* in medical apps); Figure 6 does the same for the three medical doctors involved.

In summary, nonexpert users evaluate our system better, as they are likely to appreciate the feedback provided by the app more; on the contrary, expert users might already know what to expect. The best results are obtained with the subscales *mental demand* and *effort* for patients, and with *mental demand, physical demand* and *effort* for medical doctors.

## 6 Conclusion

In this paper, we have shown the technologies and the main functionalities used for developing *reCOVeryaID*, an intelligent telemonitoring application for symptomatic, asymptomatic and pre-symptomatic COVID-19 patients. The main goal is to make it operational in Italian hospitals very soon, after final validation.

In order to make the developed prototype even more useful in an actual situation, further effort will be focused on refining its current iteration. In particular, we hope to improve through ongoing communication with the medical personnel engaged in the COVID-19 emergency as well as General Practitioners.

Furthermore, because of its ease of use and adaptability, *reCOVeryaID* can also be seamlessly applied to other domains, not limited to the COVID-19 emergency. In fact, it can be utilized to track patients with conditions like *diabetes* or *hypertension*, which broadens its scope and reinforces physicians' acceptance of it. It is obvious that adding more vital signs to the system like blood pressure and combining the various metrics in a novel way to identify potentially dangerous situations early on or even prevent them involves further utilizing artificial intelligence techniques like logical inference and knowledge representation.

## Data availability statement

The original contributions presented in the study are included in the article/Supplementary material, further inquiries can be directed to the corresponding author.

## Author contributions

DD'A: Writing – original draft, Writing – review & editing. FB: Writing – original draft, Writing – review & editing. ST: Writing – original draft, Writing – review & editing. DC: Writing – original draft, Writing – review & editing. AM: Writing – original draft, Writing – review & editing.

## Footnotes

^1^ https://flutter.dev

^2^ https://dart.dev

^3^ https://digital.ahrq.gov/health-it-tools-and-resources/evaluation-resources/workflow-assessment-health-it-toolkit/all-workflow-tools/nasa-task-load-index
