# Supplementary material for: Technologies and main functionalities of the telemonitoring application reCOVeryaID
Source: Front Big Data. 2024 Jul 22;7:1360092. doi: 10.3389/fdata.2024.1360092 (PMC11298383; doi:10.3389/fdata.2024.1360092)
Supplement: Supplementary file 1 [file Presentation_1.pdf]

Technologies and Main Functionalities of the Telemonitoring Application reCOVeryaID

Daniela D'Auria* [1], **Fabio Bettini** [2], **Selene Tognarelli** [3], **Diego Calvanese** [1,4] **and Arianna Menciassi** [3]

[1] *Free University of Bozen-Bolzano, Faculty of Engineering, Bozen-Bolzano, Italy*
[2] *University of Naples "Federico II", Department of Electrical Engineering and Information Technology, Napoli, Italy*
[3] *The BioRobotics Institute,Scuola Superiore Sant'Anna, Pisa, Italy*
[4] *Umeå University, Department of Computing Science, Umeå, Sweden*

Correspondence*:
Daniela D'Auria
daniela.dauria@unibz.it

SUPPLEMENTAL MATERIALS

This section contains screenshots of the webapp demonstrating how the various functionalities described in the paper have been implemented.

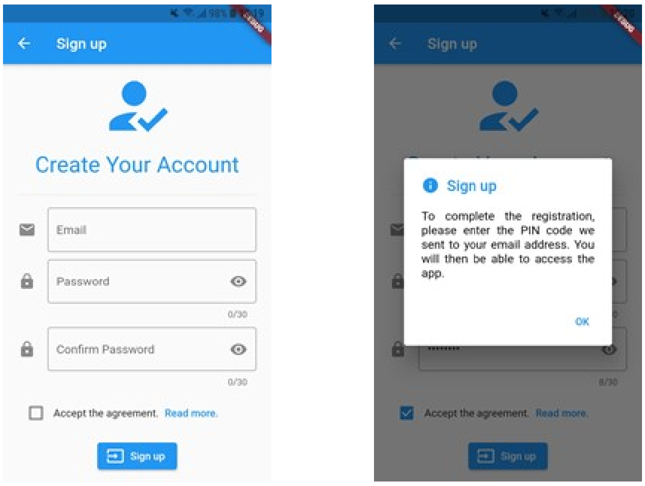

(a) (b)

Figure 1. (a) System registration and user account creation; (b) Message system registration and user account creation

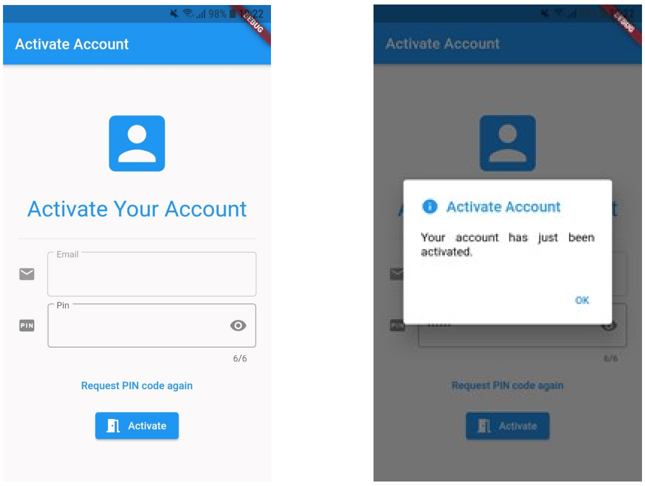

(a) (b)

Figure 2. (a) User account activation; (b) User account activation message

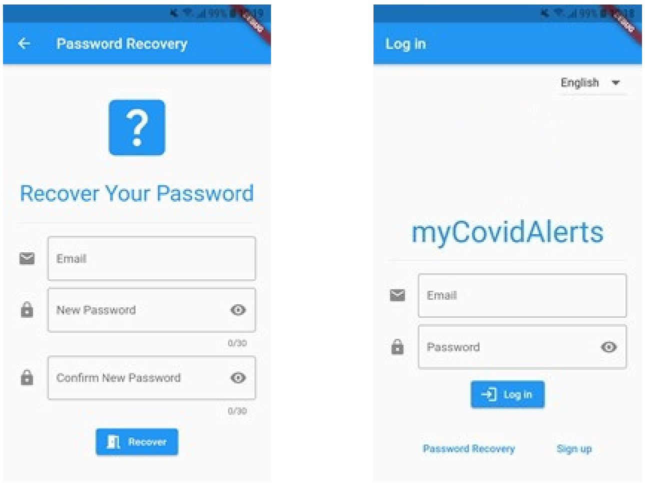

(a) (b)

Figure 3. (a) Password recovery; (b) Access

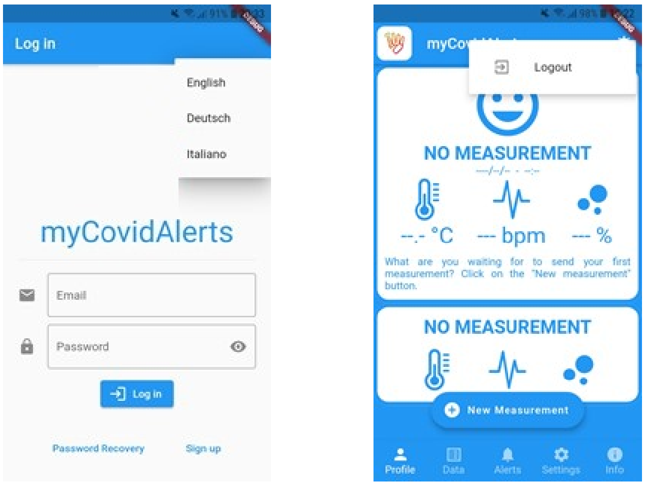

(a) (b)

Figure 4. (a) Languages; (b) Log out

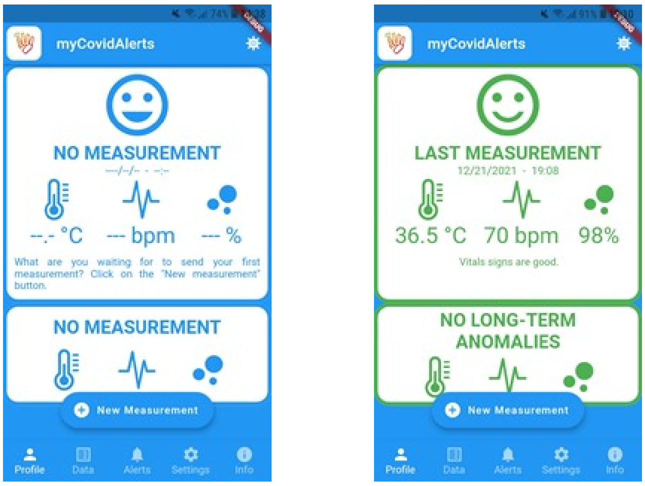

(a) (b)

Figure 5. (a) Homepage; (b) Last measurement information

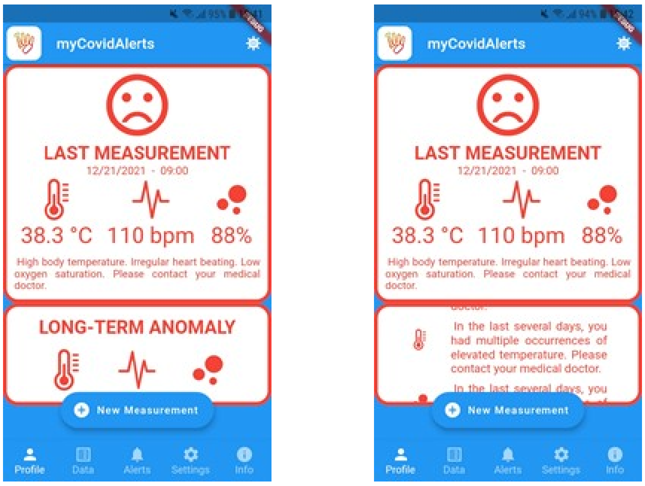

(a) (b)

Figure 6. (a) Long-term Alerts; (b) Long-term temperature alert

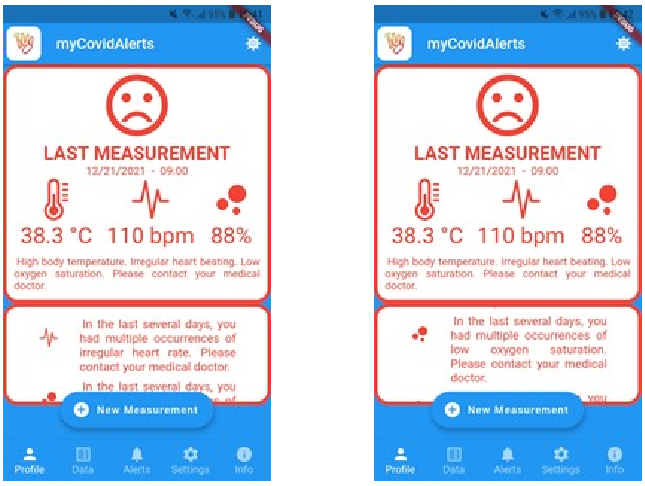

(a) (b)

Figure 7. (a) Long-term heart rate alert; (b) Long-term alert on oxygen saturation

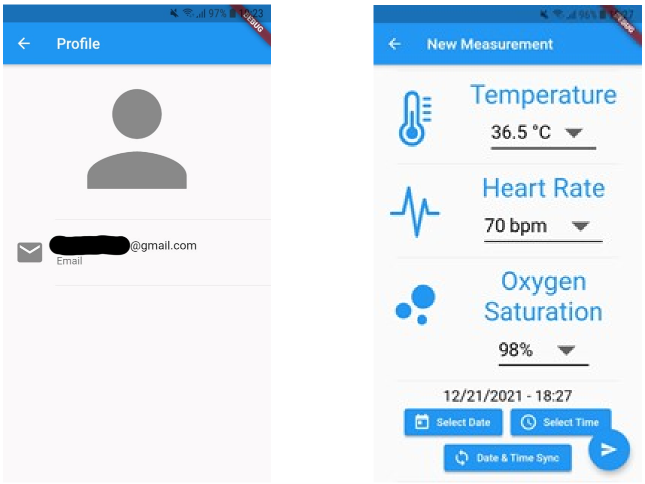

(a) (b)

Figure 8. (a) User Profile; (b) New measurement

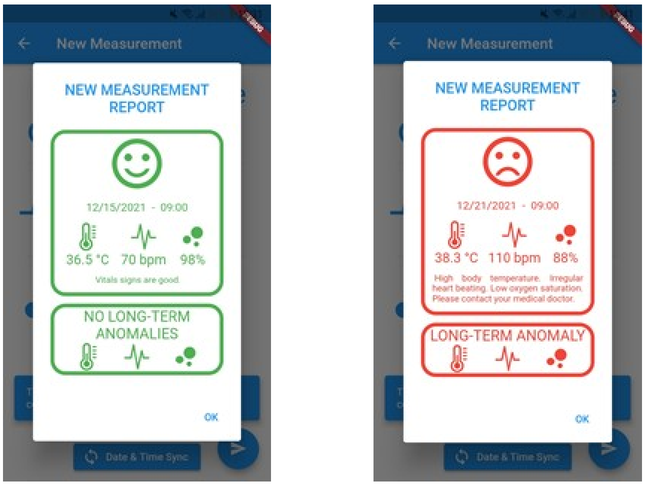

(a) (b)

Figure 9. Reports of the new measurement without long-term alerts

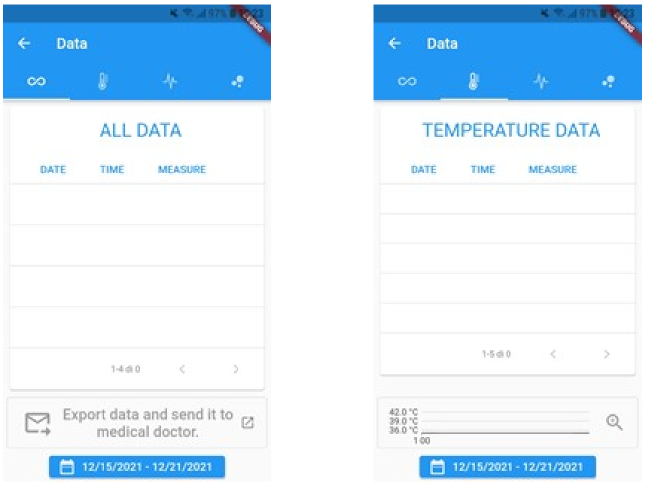

(a) (b)

Figure 10. (a) Measurement history; (b) Measurement history (temperature)

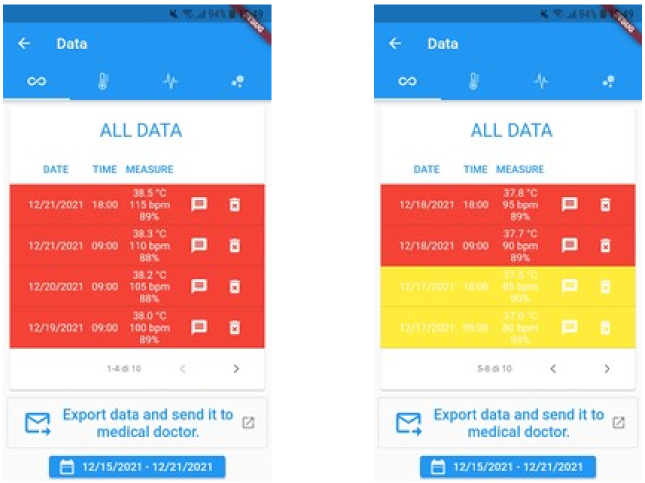

(a) (b)

Figure 11. Measurement history focused on all parameters: (a) (1 of 3); (b) (2 of 3)

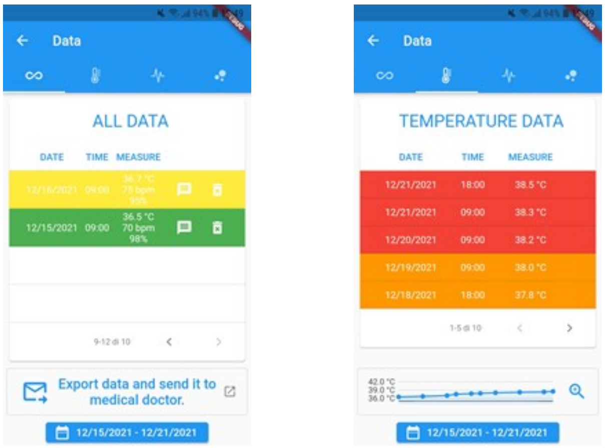

(a) (b)

Figure 12. (a) Measurement history focused on all parameters (3 of 3); (b) Temperature-focused measurement history with graphical preview (1 of 2)

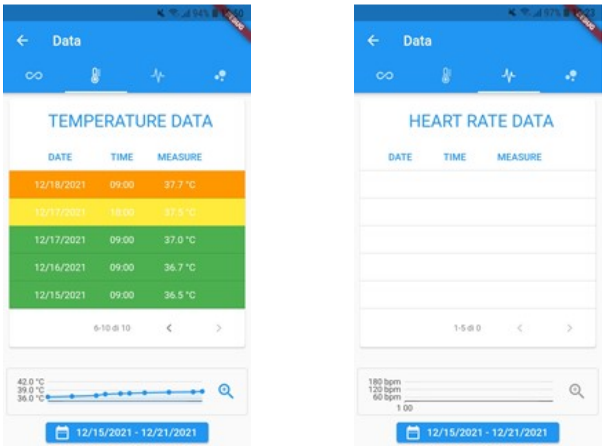

(a) (b)

Figure 13. (a) Temperature-focused measurement history with graphical preview (2 of 2); (b) Measurement history (heart rate)

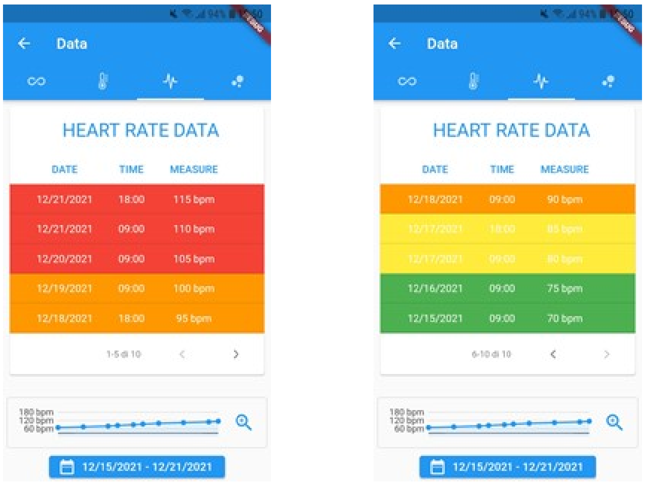

(a) (b)

Figure 14. Heart rate-focused measurement history with graphical preview: (a) (1 of 2); (b) (2 of 2)

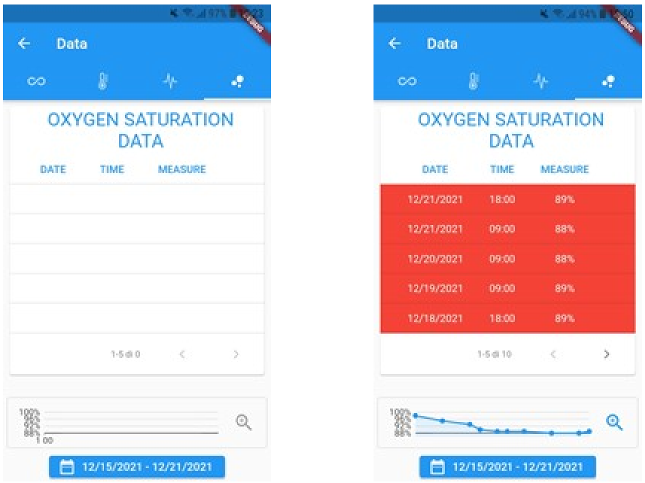

(a) (b)

Figure 15. (a) Measurement history (oxygen saturation); (b) Measurement history focused on oxygen saturation with graphical preview (1 of 2)

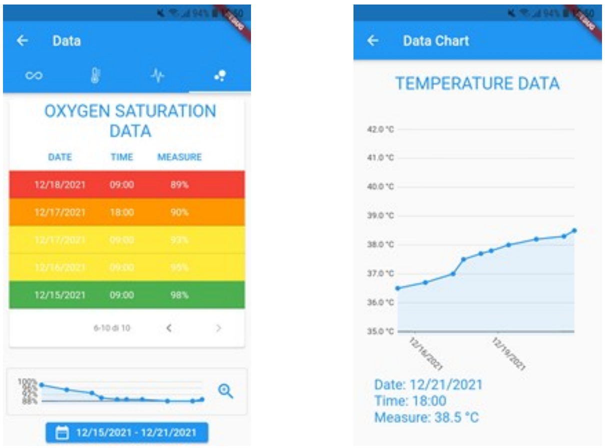

(a) (b)

Figure 16. (a) Measurement history focused on oxygen saturation with graphical preview (2 of 2); (b) Detailed temperature graph

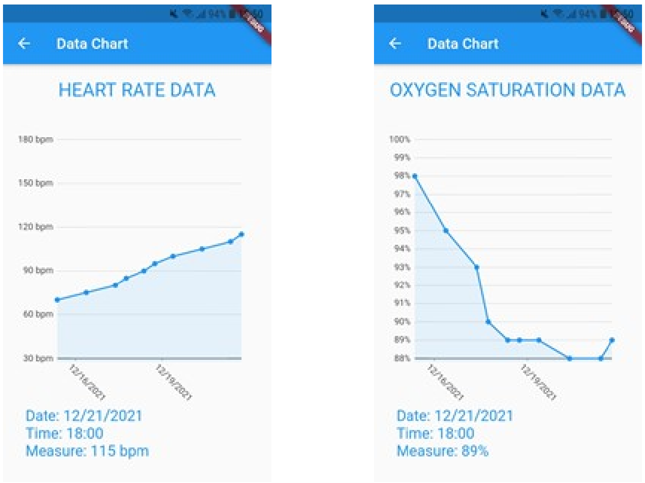

(a) (b)

Figure 17. (a) Detailed heart rate graph; (b) Detailed graph of oxygen saturation

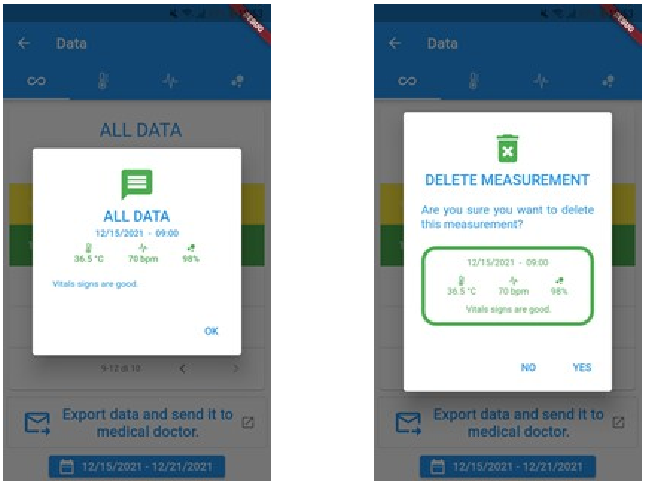

(a) (b)

Figure 18. (a) Details of the measurement; (b) Measurement cancellation

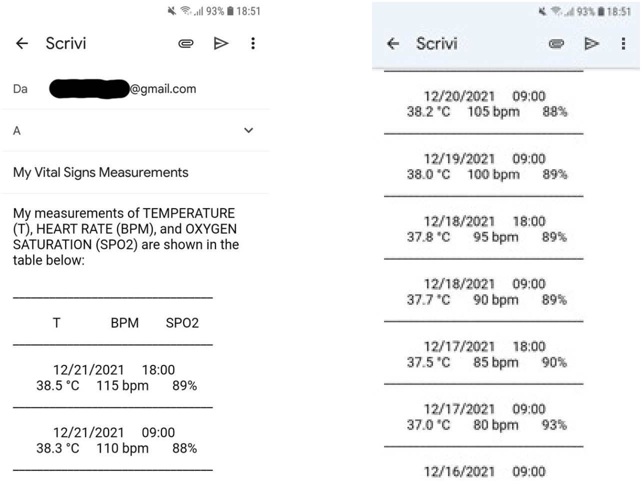

(a) (b)

Figure 19. Exporting Measurements: (a) (1 of 3); (b) (2 of 3)

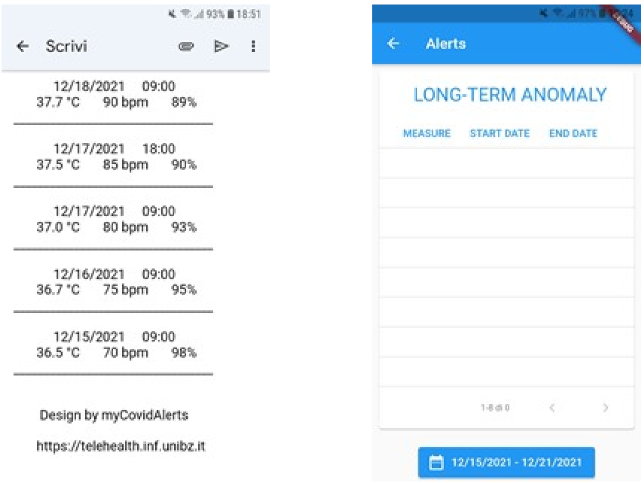

(a) (b)

Figure 20. (a) Exporting Measurements (3 of 3); (b) Long-term alert history

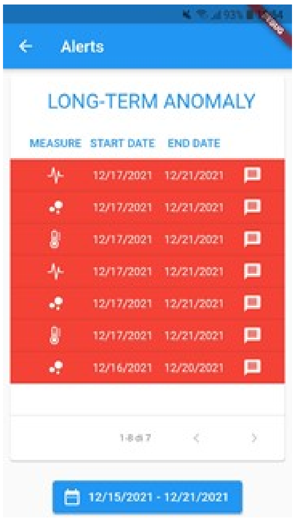

Figure 21. Long-term alert history (temperature, heart rate, oxygen saturation)

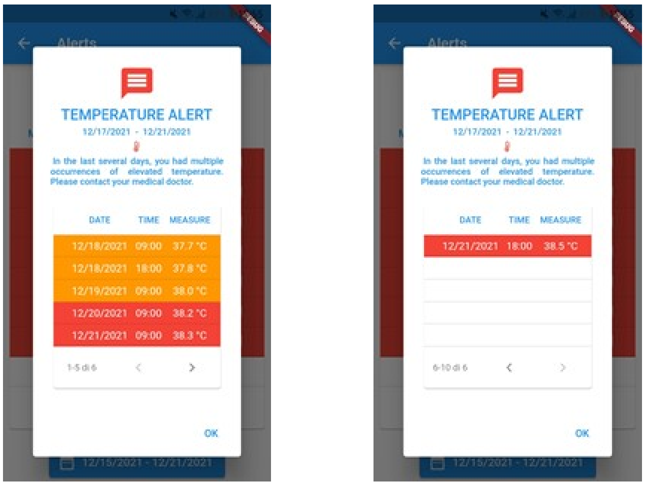

(a) (b)

Figure 22. Details of the long-term temperature alert: (a) (1 of 2); (b) (2 of 2)

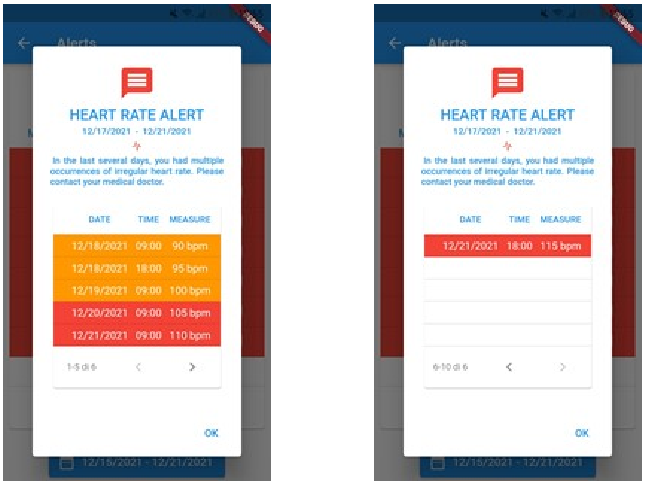

(a) (b)

Figure 23. Details of long-term heart rate alert: (a) (1 of 2); (b) (2 of 2)

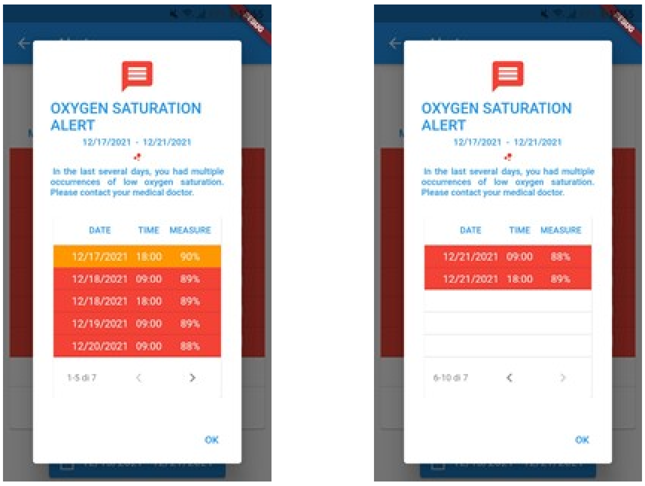

(a) (b)

Figure 24. Details of the long-term oxygen saturation alert: (a) (1 of 2); (b) (2 of 2)

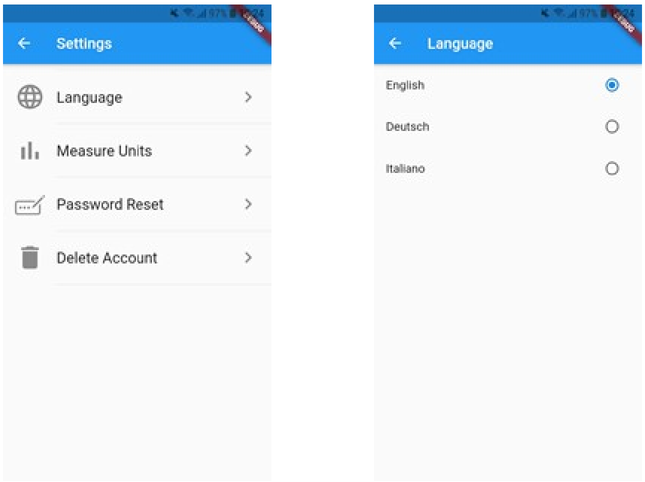

(a) (b)

Figure 25. (a) Settings; (b) Languages

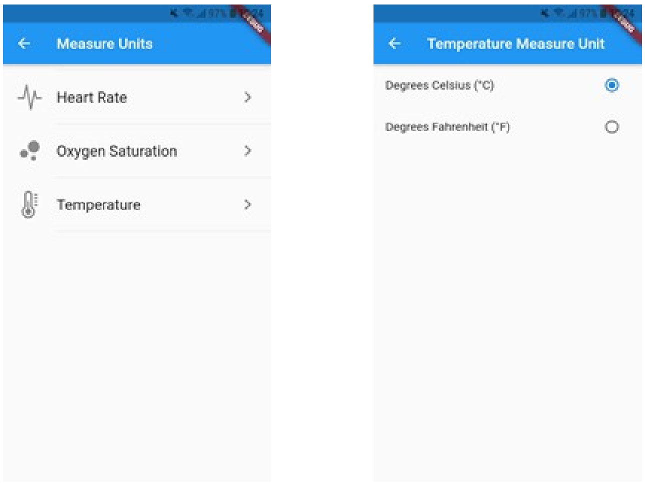

(a) (b)

Figure 26. (a) Units of measurement of parameters; (b) Temperature units of measurement

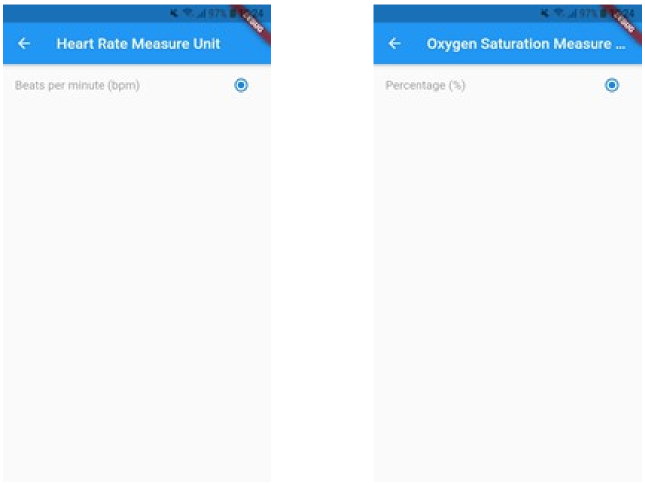

(a) (b)

Figure 27. (a) Unit of measurement of heart rate; (b) Unit of measurement of oxygen saturation

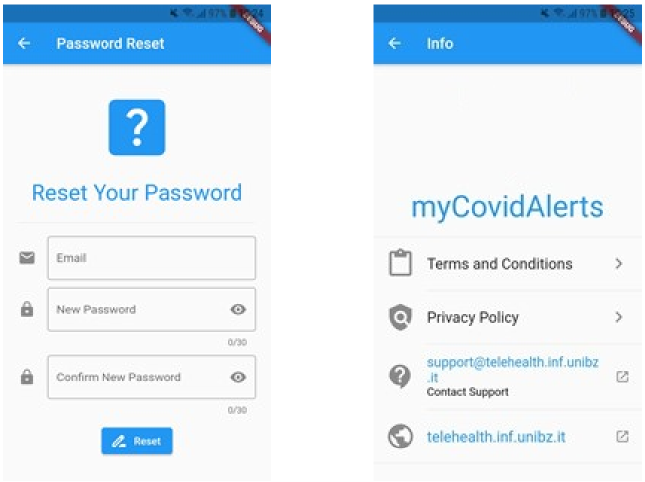

(a) (b)

Figure 28. (a) Resetting/changing your account login password; (b) Information

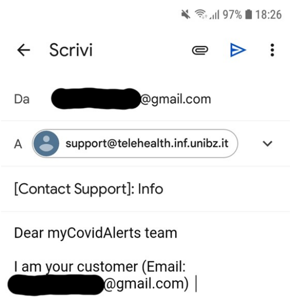

Figure 29. Contact the support team
